# Opposing effects of acute versus chronic inhibition of p53 on decitabine’s efficacy in myeloid neoplasms

**DOI:** 10.1038/s41598-019-44496-6

**Published:** 2019-06-03

**Authors:** Moe Tamura, Taishi Yonezawa, Xiaoxiao Liu, Shuhei Asada, Yasutaka Hayashi, Tomofusa Fukuyama, Yosuke Tanaka, Toshio Kitamura, Susumu Goyama

**Affiliations:** Division of Cellular Therapy, The Institute of Medical Science, The University of Tokyo, 4-6-1 Shirokanedai, Minato-ku, 108-8639 Tokyo, Japan

**Keywords:** Cancer therapy, Cell biology

## Abstract

Decitabine is a DNA methyltransferase inhibitor and is considered a promising drug to treat myelodysplastic syndromes (MDS) and acute myeloid leukemia (AML) with p53 mutations. However, whether loss of p53 in fact increases the response of MDS/AML cells to decitabine remains unclear. In this study, we assessed the role of p53 in MDS and AML cells treated with decitabine using mouse models for MLL-AF9-driven AML and mutant ASXL1-driven MDS/AML. CRISPR/Cas9-mediated depletion of p53 in MDS/AML cells did not increase, but rather decreased their sensitivity to decitabine. Forced expression of a dominant-negative p53 fragment (p53DD) in these cells also decreased their responses to decitabine, confirming that acute inhibition of p53 conferred resistance to decitabine in AML and MDS/AML cells. In contrast, MLL-AF9-expressing AML cells generated from bone marrow progenitors of *Trp53*-deficient mice were more sensitive to decitabine *in vivo* than their wild-type counterparts, suggesting that long-term chronic p53 deficiency increases decitabine sensitivity in AML cells. Taken together, these data revealed a multifaceted role for p53 to regulate responses of myeloid neoplasms to decitabine treatment.

## Introduction

p53, encoded by *TP53* in humans and *Trp53* in mice, is the most frequently mutated gene in human cancer^1,2^. p53 is a transcription factor and regulates expression of downstream target genes involved in diverse cellular processes, including apoptosis, cell cycle arrest, senescence, and metabolic regulation. In addition, p53 maintains genomic stability as the “guardian of the genome”. Through these functions, p53 plays a central role to prevent tumor initiation and progression. Loss of p53 function, either by mutation, gene deletion, or increased expression of negative regulators, leads to the development of various types of tumors, including hematopoietic neoplasms. Furthermore, p53 mutations are associated with resistance to standard chemotherapy and adverse outcomes in cancer patients.

Interestingly, recent clinical studies have shown that patients with acute myeloid leukemia (AML) and myelodysplastic syndrome (MDS) who had p53 mutations exhibited favorable responses to the treatment with decitabine^3,4^. Furthermore, clonal analyses of the decitabine-treated patients revealed the marked, but not durable, clearance of subclones with *TP53* mutations^3–5^. Decitabine is a hypomethylating agent that inhibits DNA methyltransferases (DNMTs), and is currently approved for the treatment of MDS and AML^6^. Consistent with the clinical observations, experimental studies have shown that decitabine induces cell death preferentially in p53 null or mutated cells than in p53 wild-type cells^7–9^. These findings suggest that decitabine is a promising drug to treat MDS and AML with p53 mutations. However, another report found no significant differences in the response rates of MDS patients with *TP53* mutations and those with wild-type *TP53* to hypomethylating agents^10^. In addition, several experimental studies have reported conflicting results regarding the relationship between DNA hypomethylation and p53 function. For example, loss of genomic methylation induced by *Dnmt1* depletion caused p53-dependent apoptosis in fibroblasts^11^. It was also shown that decitabine treatment provoked p53 activation and apoptosis in colon cancer cells^12^. Thus, the role of p53 in decitabine-treated tumor cells appears to be highly context-dependent. It is therefore important to determine the role of p53 in the regulation of decitabine’s efficacy using appropriate models for MDS and AML.

We have developed several mouse models for AML and MDS with MLL fusions or ASXL1 mutations. MLL fusion leukemia is an aggressive leukemia carrying chimeric fusion of the *MLL* (*KMT2A*) gene^13,14^. MLL-AF9 is one of the most prevalent forms of MLL-fusion oncogene, and has the ability to transform both human and mouse hematopoietic progenitor cells into AML cells^15^. ASXL1 is an epigenetic regulator that interacts with multiple histone modifying enzymes including BAP1 and EZH2, and is frequently mutated in MDS and AML^6,16,17^. Most *ASXL1* mutations exist in exon 12 of the gene, generating C-terminally truncated mutations. We have shown that a C-terminally truncated ASXL1 mutant promotes the development of MDS and AML in concert with NRAS, SETBP1 and RUNX1 mutations^16,18–21^.

In this study, we assessed the role of p53 in the regulation of decitabine’s efficacy using the above described mouse MDS/AML models and human cord blood cells. Our study clearly showed that acute inhibition of p53 did not increase, but rather decreased sensitivity of MDS/AML cells to decitabine. In contrast, AML cells generated from bone marrow progenitors of *Trp53*-deficient mice showed hypersensitivity to decitabine, which implies that long-term chronic inhibition of p53 function gradually increases sensitivity of AML cells to decitabine through secondary genetic and/or epigenetic changes.

## Results

### Acute inhibition of p53 confers resistance to decitabine in MLL-AF9-driven AML cells

**Figure 1:**
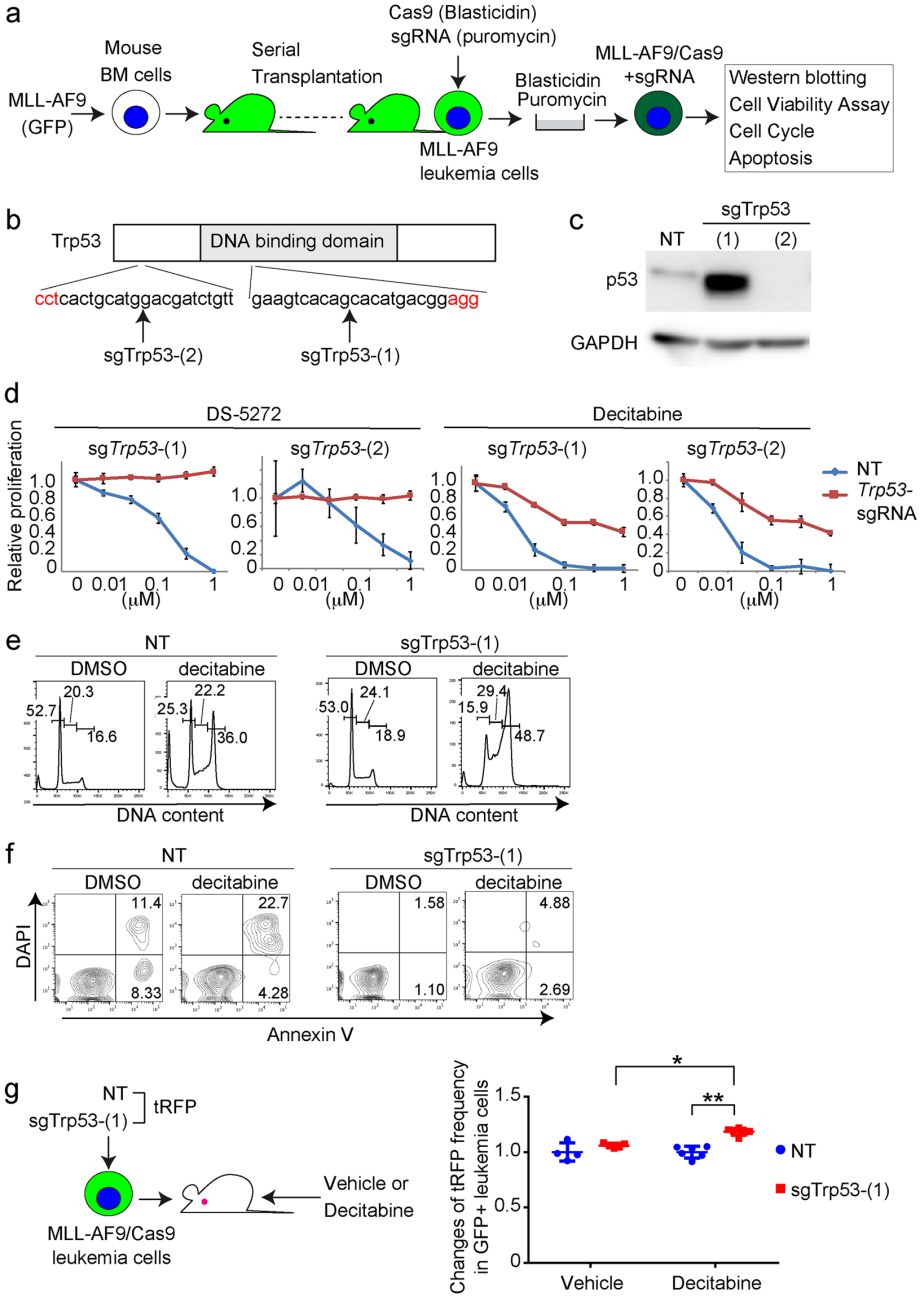
p53-depleted MLL-AF9 cells were less sensitive to decitabine. (**a**) Experimental scheme used in (**c**–**f**). Mouse bone marrow (BM) cells were transduced with MLL-AF9 (coexpress GFP) and were transplanted into recipient mice. MLL-AF9-expressing leukemia cells were harvested from spleens of moribund mice, and were serially transplanted into recipient mice. These MLL-AF9 leukemia cells were then transduced with Cas9 together with non-targeting (NT) or *Trp53*-targeting sgRNAs [sgTrp53-(1) and (2)], and were used in subsequent assays. (**b**) Schematic representation of Trp53 and sgRNA-targeting regions. (**c**) Levels of p53 protein in MLL-AF9 cells transduced with NT or *Trp53*-targeting sgRNAs. Note that p53 protein in sgTrp53-(1)-transduced cells migrated faster than that in NT-transduced cells, indicating that it is a nonfunctional mutant protein. (**d**) Cell Viability Assays were performed using Cell Counting Kit-8. The control and p53-depleted MLL-AF9 cells were treated with DS-5272 (left) or decitabine (right) at indicated concentrations for 72 h in triplicate. Data are normalized to vehicle control (0 μM group), and are shown as mean ± s.d. (**e**,**f**) Cell-cycle status (**e**) and apoptosis (**f**) were assessed after 6 or 24 hours culture with decitabine (50 nM), respectively. (**e**) FACS profiles of DMSO- or decitabine-treated MLL-AF9 cells are shown. The numbers indicate the percentages of cells in the G0/G1, S and G2/M phases. (**f**) Shown are FACS profiles of Annexin V and DAPI expression of vehicle- or decitabine-treated MLL-AF9 cells. The numbers indicate the percentages of Annexin V^+^ and Annexin V^+^DAPI^+^ cells. (**g**) MLL-AF9/Cas9 cells were transduced with NT or sgTrp53-(1) coexpressing tRFP, following transplantation into recipient mice that were treated with vehicle or decitabine (0.6 mg/kg, every ohter day). Relative ratios of the tRFP^+^ (sgRNA-transduced) fraction in GFP^+^ MLL-AF9 leukemia cells after transplantation compared with that before transplantation are shown as mean ± s.d. The frequency of sgTrp53-(1)-transduced tRFP^+^ cells was increased in mice treated with decitabine. (NT_Vehicle: n = 4, NT_Decitabine: n = 5, sgTrp53-(1)_Vehicle: n = 6, sgTrp53-(1)_Decitabine: n = 6) *p = 0.0041, **p < 0.0001. Sidak’s multiple comparisons test.

We first examined the effect of acute depletion of p53 on decitabine’s efficacy using a mouse model for MLL-AF9 leukemia. We transduced MLL-AF9 (coexpress GFP) into mouse bone marrow progenitor cells, and transplanted the cells into sublethally irradiated recipient mice. Consistent with our previous reports^15,22^, all recipient mice developed AML around 2 months after transplantation. We collected GFP^+^ leukemia cells from spleens of the moribund mice, and enriched for leukemia stem cell activity by serial transplantation through secondary, tertiary, and quaternary recipient mice. These MLL-AF9-expressing cells with strong leukemogenicity were then transduced with Cas9 together with a non-targeting (NT)-single guide RNA (sgRNA) and two independent sgRNAs targeting *Trp53* (Fig. 1a,b). sgTrp53-(2) induced nearly complete depletion of p53 protein, while sgTrp53-(1) induced expression of aberrant p53 protein that migrated faster than wild-type p53 protein in MLL-AF9 cells (Fig. 1c). MLL-AF9 cells transduced with the *Trp53-*targeting sgRNAs became resistant to a p53-Mdm2 interaction inhibitor DS-5272^23^ (Fig. 1d), indicating that both sgRNAs depleted functional p53. We then compared sensitivity of MLL-AF9 cells transduced with these sgRNAs to decitabine using Cell Viability Assay. Decitabine inhibited the growth of control MLL-AF9 cells more efficiently than that of p53-depleted MLL-AF9 cells (Fig. 1d).

To characterize the decitabine-induced suppression of cell growth, we analyzed cell cycle status and apoptosis in vehicle- and decitabine-treated MLL-AF9 cells. Decitabine treatment induced G2/M arrest in both p53-intact and p53-depleted MLL-AF9 cells (Fig. 1e), suggesting that p53 is dispensable for decitabine-mediated cell cycle arrest. However, decitabine-induced cell death was not evident in p53-depleted MLL-AF9 cells (Fig. 1f), which probably accounts for their reduced response to decitabine.

To assess the role of p53 on decitabine’s efficacy *in vivo*, we then transduced tRFP-coexpressing sgRNAs [NT and sgTrp53-(1)] into MLL-AF9/Cas9 cells. These MLL-AF9/Cas9/sgRNA-tRFP cells were transplanted into recipient mice, and the mice were treated with vehicle or decitabine. Although *Trp53*-depletion did not alter leukemic progression of MLL-AF9 cells in non-treated mice, it decreased sensitivity of MLL-AF9 cells to decitabine, as evidenced by the increase of *Trp53*-depleted (tRFP^+^) cells in decitabine-treated mice (Fig. 1g). Thus, acute inhibition of p53 function by CRISPR/Cas9-mediated *Trp53* depletion reduced responsiveness of MLL-AF9 cells to decitabine both *in vitro* and *in vivo*.

**Figure 2:**
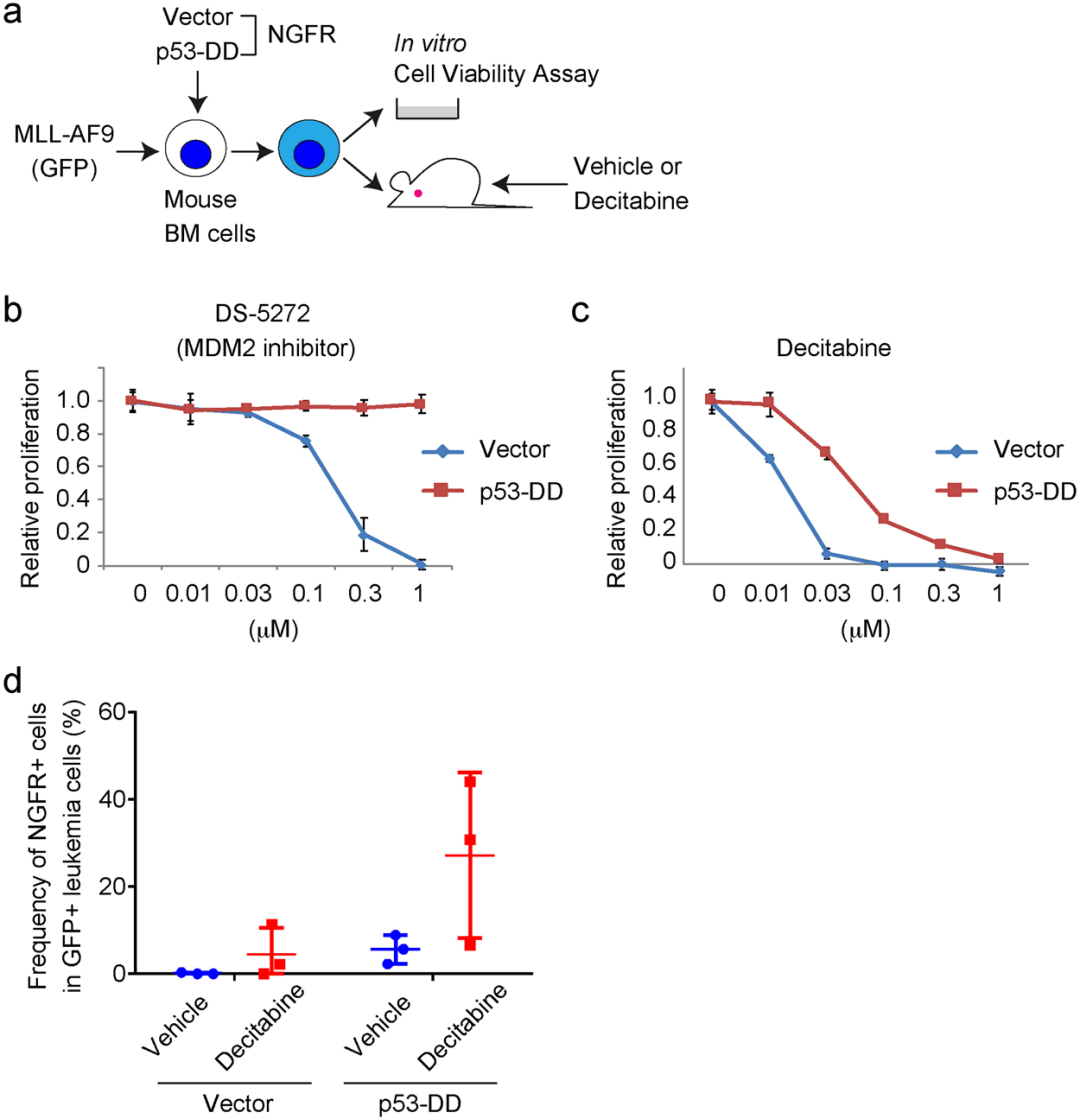
p53DD-transduced MLL-AF9 cells were less sensitive to decitabine. (**a**) Experimental scheme used in (**b**–**d**). Mouse bone marrow (BM) cells were transduced with MLL-AF9 (coexpress GFP) and p53DD (coexpress NGFR), and were cultured *in vitro* or transplanted into recipient mice. (**b**,**c**) Cell Viability Assay using vector or p53DD-transduced MLL-AF9 cells, treated with DS-5272 (**b**) or decitabine (**c**) at indicated concentrations for 72 h in triplicate. Data are normalized to vehicle control (0 μM group), and are shown as mean ± s.d. (**d**) Mouse BM cells were retrovirally transduced with MLL-AF9 (coexpress GFP) together with vector or p53DD (coexpress NGFR), and the cells were transplanted into mice. Frequencies of the NGFR^+^ (vector/p53DD-transduced) fraction in GFP^+^ (MLL-AF9-transduced) leukemia cells after transplantation are shown. The frequency of p53DD-transduced cells were increased only in mice treated with decitabine (N = 3 per group).

Next, we transduced vector or a dominant-negative p53 fragment (p53DD^24^) (each coexpresses NGFR) into MLL-AF9 cells and assessed the effect of decitabine on these cells both *in vitro* and *in vivo* (Fig. 2a). MLL-AF9 cells transduced with p53DD grew normally in the presence of DS-5272 (Fig. 2b), indicating the efficient inhibition of p53 function by p53DD in them. Similar to the results of p53-depletion, p53DD-transduced cells were relatively resistant to decitabine compared with vector-transduced cells (Fig. 2c). We then transplanted vector or p53DD-transduced MLL-AF9 cells into recipient mice, and treated these mice with vehicle or decitabine. Flow cytometric analysis of NGFR^+^ (vector/p53DD-transduced) cells in peripheral blood at day 16 revealed a tendency of increase of p53DD-transduced cells only in decitabine treated mice (Fig. 2d). These data suggest that forced expression of p53DD also conferred resistance to decitabine in MLL-AF9 cells, as *Trp53* depletion did.

### Acute inhibition of p53 confers resistance to decitabine in mouse MDS/AML cells and human cord blood cells

**Figure 3:**
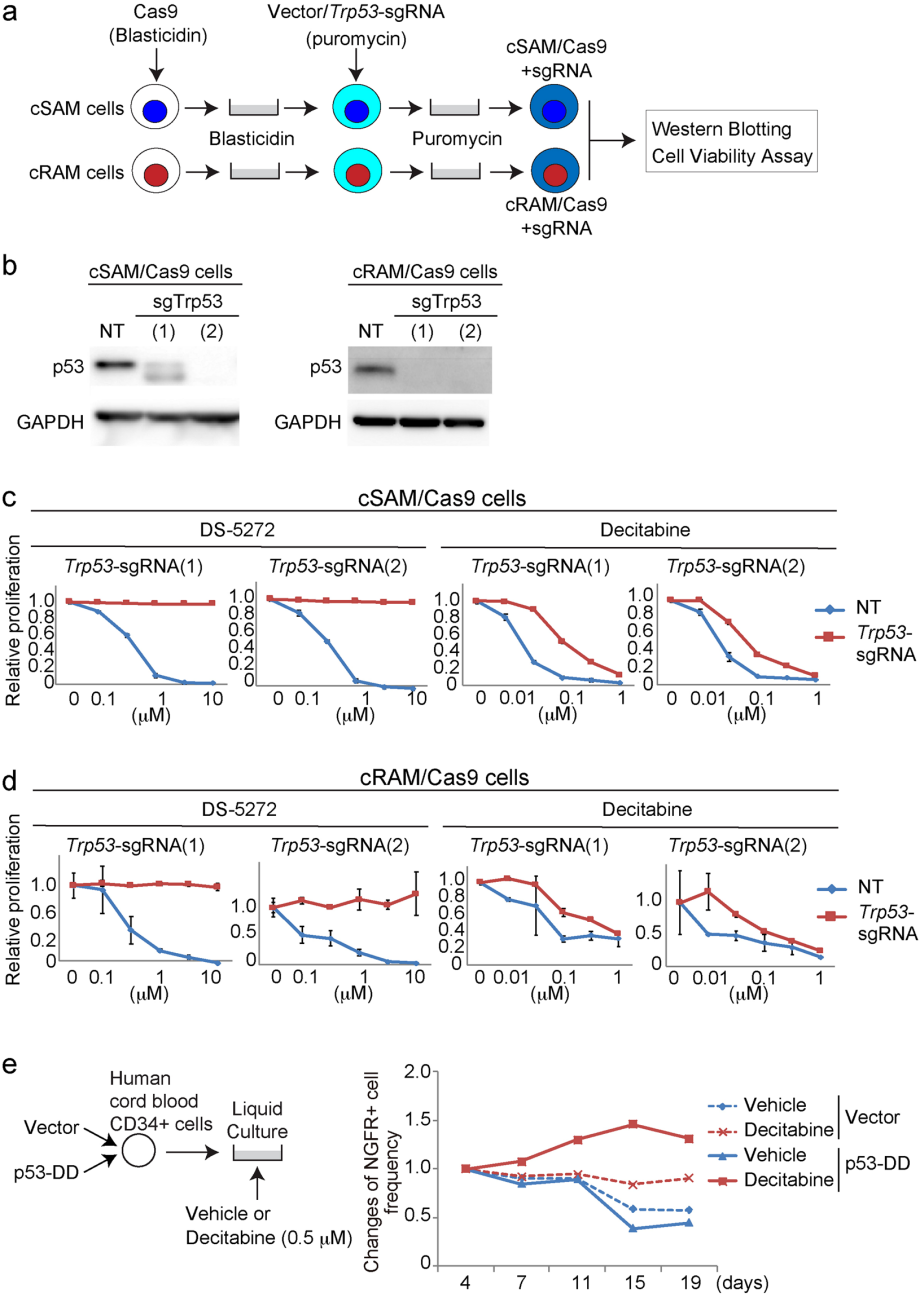
p53 inhibition conferred resistance to decitabine in cSAM, cRAM and CB cells. (**a**) Experimental scheme used in (**b–d**). cSAM cells^21^ and cRAM cells^19^ were transduced with Cas9 together with non-targeting (NT) or *Trp53*-targeting sgRNAs [sgTrp53-(1) and (2)], and were used in subsequent assays. (**b**) Levels of p53 protein in cSAM and cRAM cells transduced with NT or *Trp53*-targeting sgRNAs. (**c**,**d**) Cell Viability Assay using control and p53-depleted cSAM (**c**) or cRAM (**d**) cells, treated with DS-5272 or decitabine at indicated concentrations for 72 h in triplicate. Data are normalized to vehicle control (0 μM group), and are shown as mean ± s.d. (**e**) Human cord blood (CB) CD34^+^ cells were transduced with a vector control or p53DD (coexpress NGFR). The cells were cultured in cytokine containing media to monitor the changes of NGFR frequency. p53DD showed the growth-promoting effect in CB cells only in the presence of decitabine in culture.

We have generated two mouse MDS/AML cell lines expressing ASXL1 mutations; cSAM cells and cRAM cells. cSAM (combined expression of SETBP1 and ASXL1 Mutations) cells were generated by combined expression of two MDS-associated mutations; SETBP1-D868N and ASXL1-E635RfsX15, in mouse bone marrow progenitor cells^20,21^. cRAM (combined expression of RUNX1 and ASXL1 Mutations) cells were generated by transducing a C-terminally truncated RUNX1 mutant (RUNX1-S291fsX300^25^) into bone marrow progenitors derived from the conditional knock-in mice expressing mutant Asxl1 mimicking human ASXL1-E635RfsX15 mutation^19,21^. To examine the effect of p53 inhibition on the growth of MDS/AML cells, we transduced Cas9 and *Trp53*-targeting sgRNAs into cSAM and cRAM cells (Fig. 3a). Both cSAM and cRAM cells transduced with sgTrp53-(1) and (2) did not express full-length p53 (Fig. 3b), and were completely resistant to DS-5272 (Fig. 3c,d), indicating the efficient depletion of functional p53 in them. Similar to the results of MLL-AF9 cells, p53-depleted cSAM and cRAM cells became relatively resistant to decitabine (Fig. 3c,d). We then assessed the relationship between p53 and decitabine in human cord blood CD34^+^ cells. We transduced vector or p53DD (each coexpresses NGFR) into cord blood CD34^+^ cells, and cultured these cells in the absence or presence of decitabine. We observed increase of p53DD-transduced NGFR^+^ cells only in the culture containing decitabine (Fig. 3e), indicating again the important role of p53 to enhance the growth-inhibitory effect of decitabine. Thus, acute inhibition of p53 conferred resistance to decitabine in both normal and malignant hematopoietic cells.

### AML cells generated from bone marrow progenitors of *Trp53*(−/−) mice show hypersensitivity to decitabine

**Figure 4:**
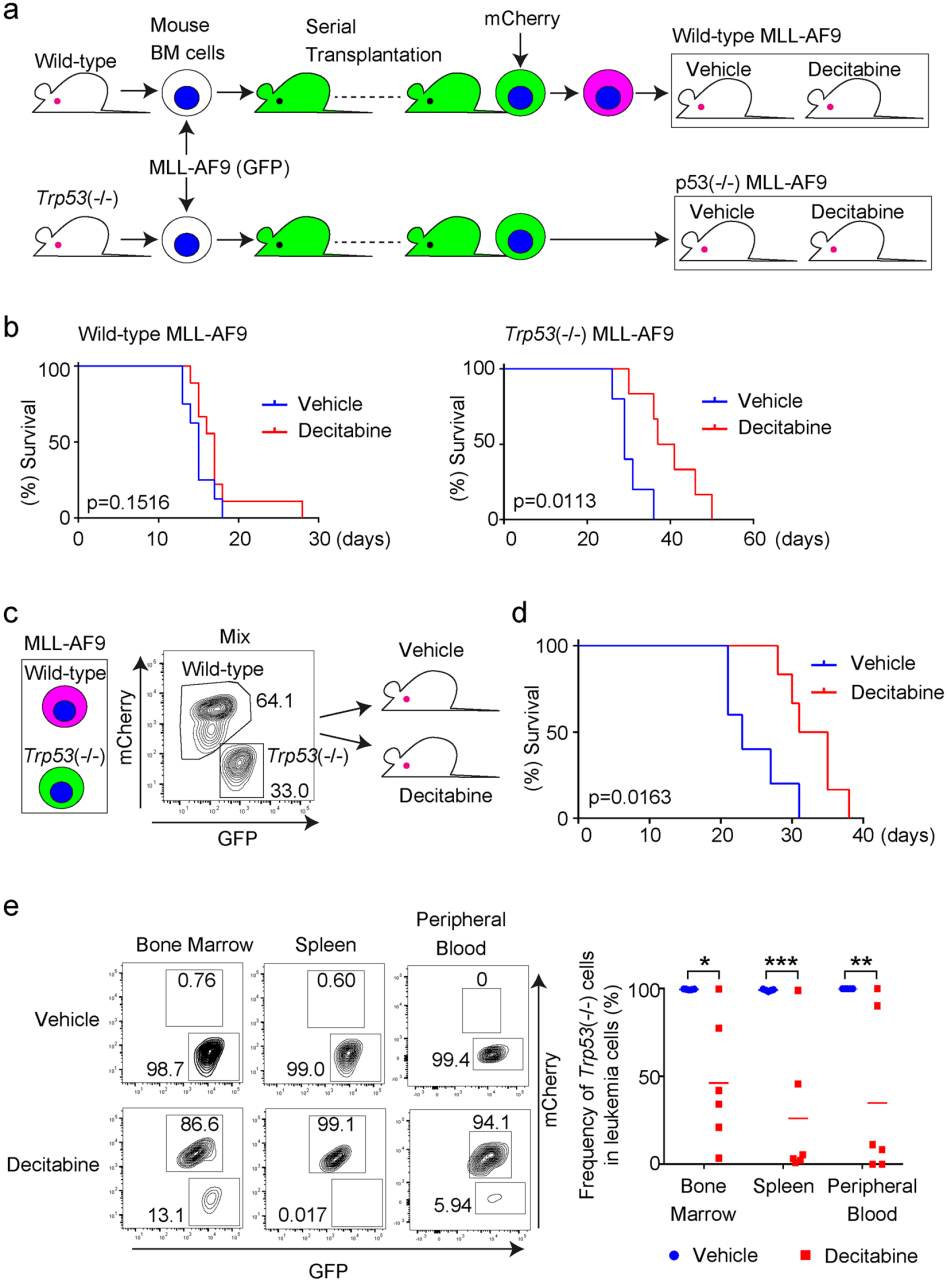
MLL-AF9 cells generated from *Trp53*(−/−) BM cells were sensitive to decitabine. (**a**) Experimental scheme used in (**b**). Mouse bone marrow (BM) cells derived from wild-type or *Trp53*(−/−) mice were transduced with MLL-AF9 (coexpress GFP) and were transplanted into recipient mice. MLL-AF9-expressing leukemia cells were harvested from spleens of moribund mice, and were serially transplanted into recipient mice. Wild-type and *Trp53*(−/−) MLL-AF9 cells were then transplanted into recipient mice following subcutaneous administration of decitabine (0.6 mg/kg) to them. (**b**) (left) Overall survival of mice that received wild-type MLL-AF9 cells. N = 8 (vehicle) or 9 (decitabine). (right) Overall survival of mice that received *Trp53*(−/−) MLL-AF9 cells. N = 5 (vehicle) or 6 (decitabine). P value was calculated using a log-rank test. (**c**) Experimental scheme used in (**d**,**e**). Wild-type and *Trp53*(−/−) MLL-AF9 cells were mixed before transplantation and were injected into recipient mice following subcutaneous administration of decitabine (0.6 mg/kg) to them. (**d**) Overall survival of mice that received wild-type and *Trp53*(−/−) MLL-AF9 cells. N = 5 (vehicle) or 6 (decitabine). P value was calculated using a log-rank test. (**e**) The frequency of wild-type (mCherry^+^) and *Trp53*(−/−) (GFP^+^) MLL-AF9 cells was assessed in bone marrow, spleen, and peripheral blood 16 days after transplantation. Note the substantial reduction of the frequency of *Trp53*(−/−) cells in mice treated with decitabine. (right) Representative FACS plots. (left) Percentages of *Trp53*-deficient (GFP^+^ only) MLL-AF9 cells in all (both GFP^+^ and mCherry^+^) MLL-AF9 leukemia cells. N = 5 (vehicle) or 6 (decitabine). *P = 0.0303, **P = 0.0152, ***P = 0.0087, Mann-Whitney U test.

Several studies have revealed phenotypic differences between transient and long-term depletion of particular genes in cells, partly due to genetic compensation in response to gene knockout^26^. To assess the influence of chronic p53 deficiency in AML cells, we next established a mouse AML model using *Trp53*-deficient mice. Because a compensatory network for p53 deficiency should have been established during embryonic development, cells derived from *Trp53*-deficient mice are suitable for this purpose. We transduced MLL-AF9 (coexpress GFP) into bone marrow progenitor cells derived from wild-type or *Trp53*(−/−) mice to generate MLL-AF9 leukemia cells through serial transplantations. We then transplanted these wild-type and *Trp53*-deficient MLL-AF9 leukemia cells into recipient mice, and treated them with vehicle or decitabine (Fig. 4a). Interestingly, in contrast to earlier results, decitabine treatment significantly prolonged survival of mice that received *Trp5*3-deficient MLL-AF9 cells, while it showed only a marginal effect on those receiving wild-type MLL-AF9 cells (Fig. 4b). To directly compare sensitivity of wild-type and *Trp53*-deficient MLL-AF9 cells to decitabine, we next performed a competitive transplantation assay. We transduced mCherry into wild-type MLL-AF9 cells as a marker, mixed wild-type (mCherry^+^) and *Trp53*-deficient (GFP^+^) MLL-AF9 cells, and transplanted them to recipient mice. The recipient mice who received both wild-type (mCherry^+^) and *Trp53*-deficient (GFP^+^) MLL-AF9 cells were then treated with vehicle or decitabine (Fig. 4c). Again, treatment with decitabine prolonged survival of these recipient mice (Fig. 4d). *Trp53*-deficient (GFP^+^) cells always became the major population in mice treated with vehicle control, indicating their stronger leukemogenic activity than wild-type counterparts. On the other hand, the ratio of GFP^+^/mCherry^+^ cells was highly variable between individual mice, and importantly, wild-type MLL-AF9 (mCherry^+^) cells often became dominant in decitabine-treated mice (Fig. 4e). These data suggest the increased *in vivo* sensitivity of *Trp53*-deficient MLL-AF9 cells to decitabine. Thus, contrary to the effect of acute p53 inhibition, chronic inhibition of p53 function appears to increase sensitivity of AML cells to decitabine treatment.

### Diverse effects of acute and chronic p53 inhibition in MLL-AF9 leukemia cells

**Figure 5:**
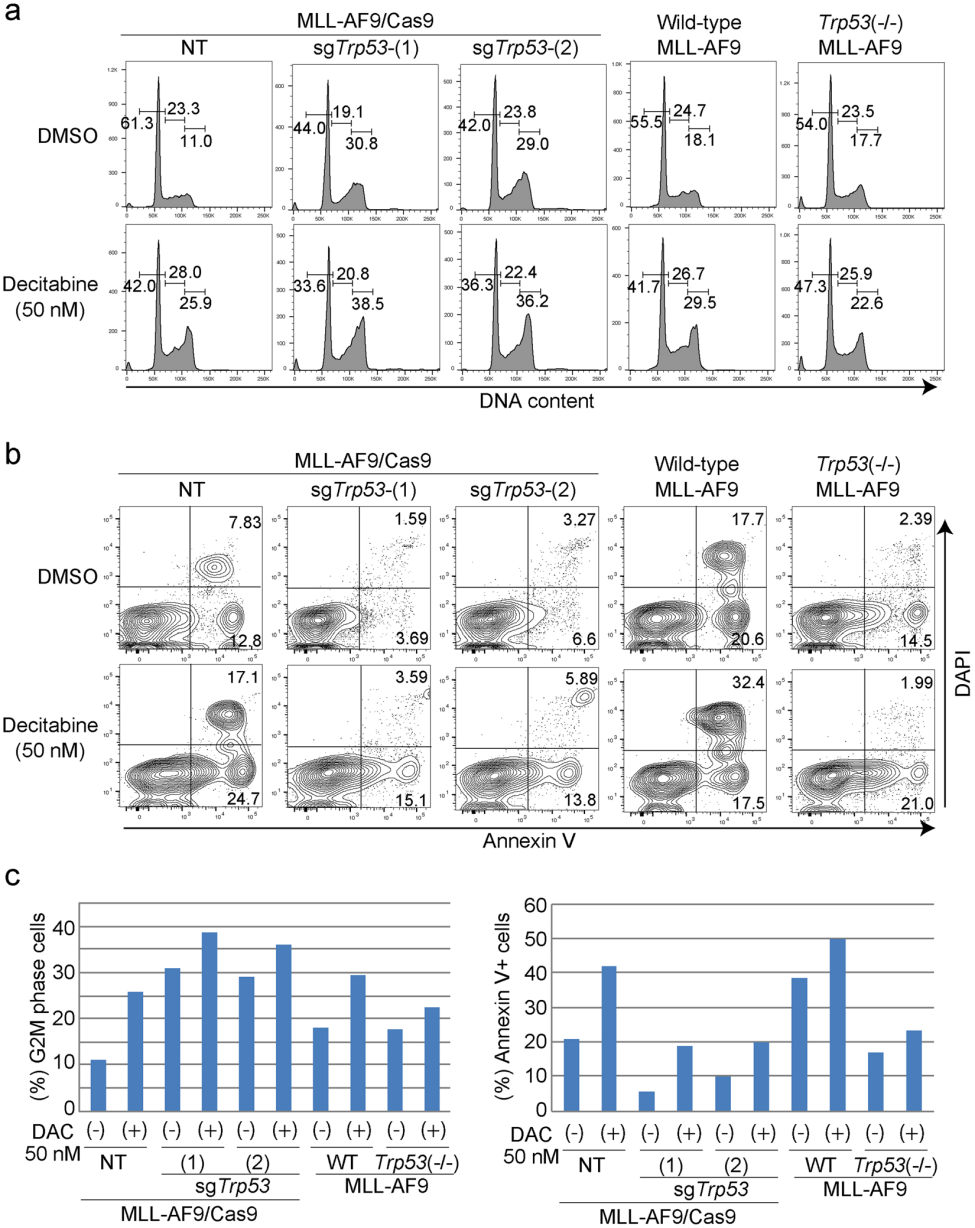
Effects of acute and chronic p53 depletion on cell cycle and apoptosis in MLL-AF9 leukemia cells. (**a–c**) Cell-cycle status (**a**) and apoptosis (**b**) were assessed after 6 or 24 hours culture with vehicle or decitabine (50 nM), respectively. (**a**) FACS profiles of DMSO- or decitabine-treated MLL-AF9 cells are shown. The numbers indicate the percentages of cells in the G0/G1, S and G2/M phases. (**b**) FACS profiles of Annexin V and DAPI expression of DMSO- or decitabine-treated MLL-AF9 cells are shown. The numbers indicate the percentages of Annexin V^+^ and Annexin V^+^DAPI^+^ cells. (**c**) The frequency of G2/M phase cells in (**a**) (left) and that of Annexin V^+^ cells in (**b**) are shown as bar graphs.

**Figure 6:**
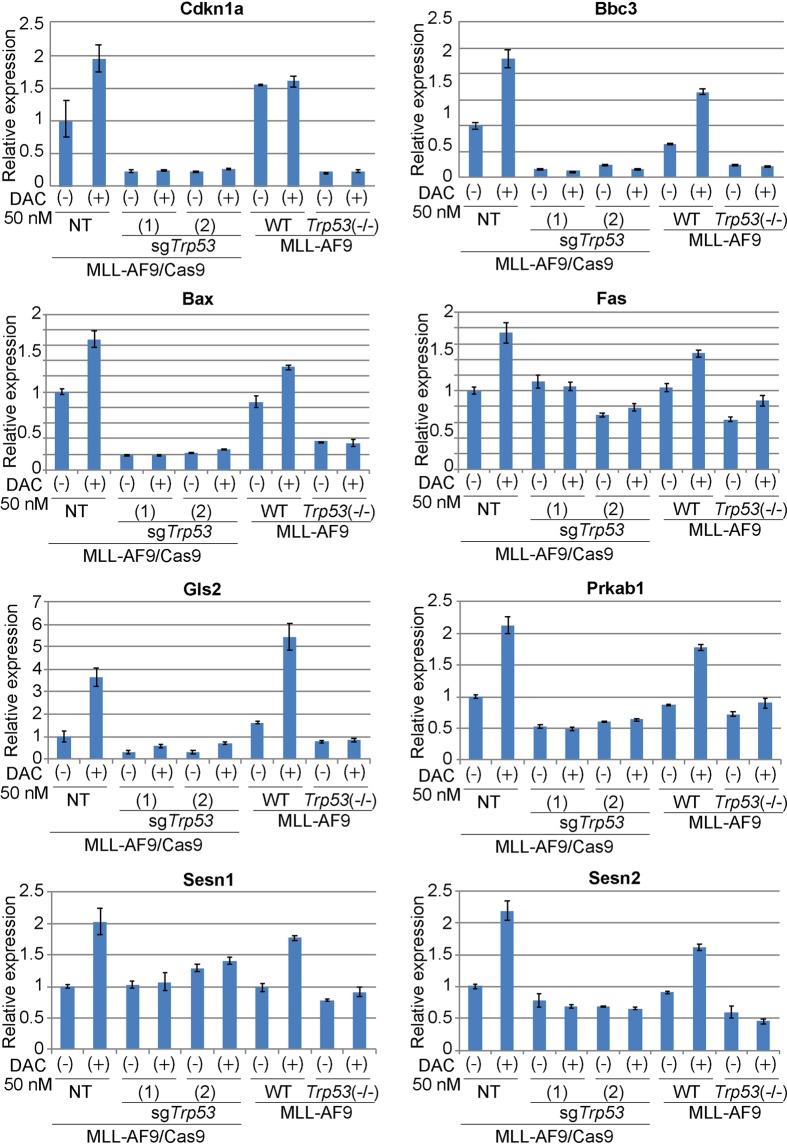
Effects of acute and chronic p53 depletion on expression of p53 target genes in MLL-AF9 leukemia cells. Quantitative PCR of relative mRNA levels for several p53 target genes in MLL-AF9 cells with indicated p53 modulations. Cells were treated with DMSO or decitabine (50 nM) for 24 hours. Results were normalized to Gapdh, with the relative mRNA level in NT-transduced control cells was set to 1. Data are shown as mean ± s.d. of duplicate wells.

Next, we compared the effect of acute and chronic p53 depletion on cell cycle, apoptosis, differentiation and expression of p53-target genes in control and decitabine-treated MLL-AF9 cells. Consistent with earlier results, decitabine induced G2/M arrest in MLL-AF9 cells irrespective of p53 status (Fig. 5a,c). Decitabine also induced apoptosis in p53-intact MLL-AF9 cells, while the decitabine-provoked apoptosis was markedly attenuated in both sgTrp53-transduced and *Trp53*-deficient MLL-AF9 cells (Fig. 5b,c). In addition, decitabine treatment resulted in upregulation of multiple p53-target genes^27^ involved in cell cycle (Cdkn1a), apoptosis (Bbc3, Bax, Fas), metabolism (Gls2, Prkab1), and translation (Sesn1, Sesn2) (Fig. 6). As expected, these decitabine-induced expression changes were not observed in MLL-AF9 cells with CRISPR/Cas9-mediated *Trp53* depletion and germline *Trp53* deficiency. Thus, both acute and chronic p53 deficiency inhibit the decitabine-induced activation of canonical p53 effector pathways.

**Figure 7:**
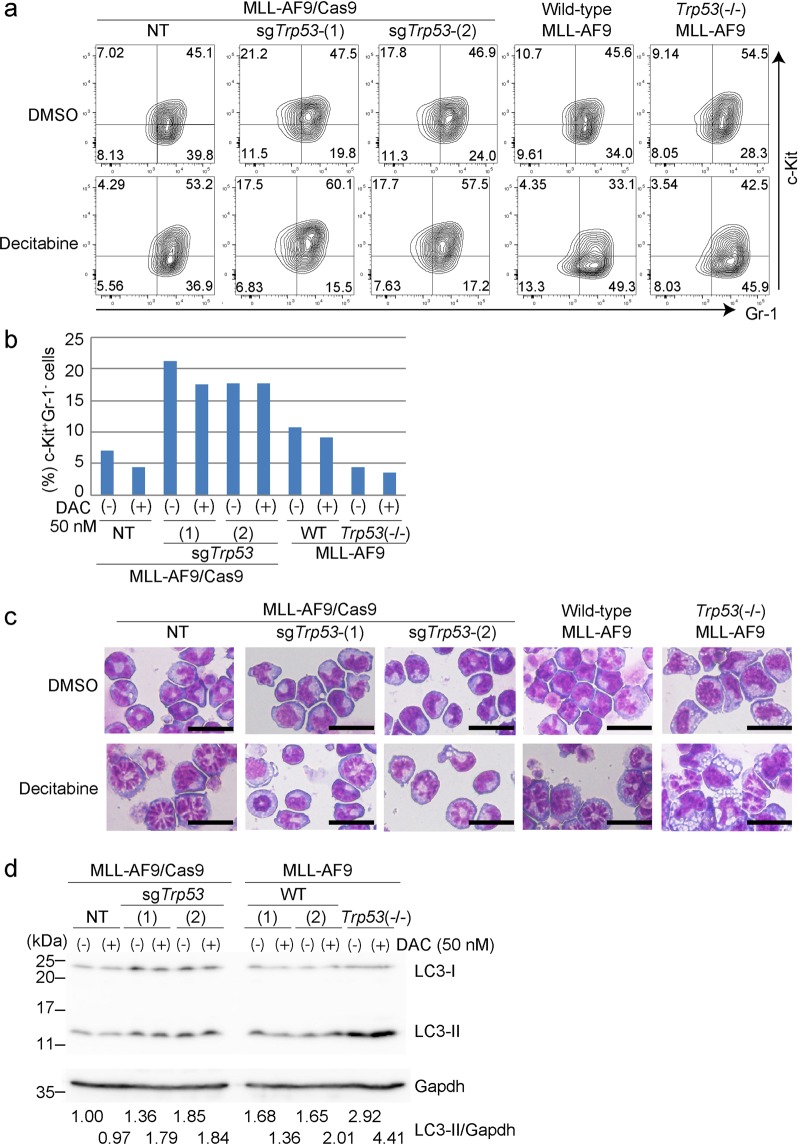
Effects of acute and chronic p53 depletion on differentiation and autophagy in MLL-AF9 leukemia cells. Immunophenotypes (**a**,**b**), morphology (**c**) and autophagy (**d**) were assessed after 24 hours culture with vehicle or decitabine (50 nM). (**a**) FACS profiles of Gr-1 and c-Kit expression of vehicle- or decitabine-treated MLL-AF9 cells with the indicated p53 modulations are shown. The numbers indicate the percentages of cells in each gate. (**b**) The frequencies of c-Kit^+^Gr-1^−^ LSC-enriched population in each MLL-AF9 culture are shown as a bar graph. (**c**) Wright-Giemsa staining of cytospin preparations of each MLL-AF9 culture. Scale bars; 25 μm. (**d**) Levels of LC3 and Gapdh proteins in each MLL-AF9 culture. Band intensities of LC3-II relative to Gapdh are shown. The value of LC3-II/Gapdh in NT-transduced cells without decitabine treatment was set to 1.

We then examined immunophenotypes of these MLL-AF9 cells. Acute p53 depletion using CRISPR/Cas9 resulted in an increase of immature cell populations (c-Kit^+^Gr-1^−^) that are enriched for self-renewing leukemia stem cells (LSCs)^28^. Interestingly, the c-Kit^+^Gr-1^−^ LSC-enriched population was not increased in *Trp53*-deficient MLL-AF9 cells (Fig. 7a,b). Although decitabine induced differentiation of all these MLL-AF9 cells (Fig. 7a–c), the distinct immunophenotypes between sgTrp53-transduced and *Trp53*-deficient MLL-AF9 cells in steady state could underlie the opposing effects of acute vs chronic p53 depletion on decitabine’s efficacy *in vivo*. We also assessed expression of LC3-II, a marker of autophagy^29^, in vehicle or decitabine-treated MLL-AF9 cells. The LC3-II expression was highest in *Trp53*-deficient MLL-AF9 cells, which was further increased by decitabine treatment (Fig. 7d). These data suggest that chronic p53 deficiency may lead to increased autophagy signaling, which could also contribute to the enhanced sensitivity of *Trp53*-deficient MLL-AF9 cells to decitabine.

## Discussion

Development of drugs that target p53-mutated tumor cells has been an important challenge. Decitabine is a promising candidate with such function. However, it is still under debate whether loss of p53 in fact increases the response of tumor cells to decitabine. Our data strongly indicate that acute inhibition of p53 does not increase sensitivity, but rather confers resistance to decitabine in MDS and AML cells. Therefore, co-inhibition of DNMTs and p53 will not be a therapeutic option for p53-intact tumors. Instead, combination therapy with hypomethylating agents and p53 activators could show synergistic inhibitory effects in myeloid neoplasms with wild-type p53.

In contrast to the effect of acute p53 inhibition, long-term deficiency of p53 appears to increase sensitivity of AML cells to decitabine. We found that MLL-AF9-driven AML cells generated from bone marrow progenitor cells of *Trp53*(−/−) mice were more sensitive to decitabine treatment than their p53-intact counterparts *in vivo*. Consistent with our data, a previous report also showed that decitabine induced differentiation of MLL-AF9 cells through p53-independent mechanisms^30^. Thus, these findings suggest that AML cells with chronic p53 deficiency/mutations retain sensitivity, or even become more sensitive to decitabine treatment.

Mechanisms for the phenotypic differences between acute and chronic inhibition of p53 in MDS/AML cells warrant further investigation. Several recent studies have revealed genetic compensation in response to gene knockout can explain the different results obtained by acute and chronic inhibition of a specific gene^26^. For example, conditional depletion of tumor suppressor *RB1* enables cell-cycle reentry of quiescent MEFs, while quiescent MEFs derived from *Rb1*-mutant mice are unable to reenter the cell cycle, partly due to the compensatory upregulation of p107^31^. Similar genetic compensation may occur in AML cells generated from *Trp53*(−/−) bone marrow cells. In our MLL-AF9 leukemia model, acute p53 depletion using CRISPR/Cas9 increased the frequency of LSC-enriched population, whereas chronic p53 deficiency in MLL-AF9 cells rather decreased the LSC fraction. We also found the substantial enhancement of autophagy signaling only in MLL-AF9 cells with chronic p53 deficiency. These differences between acute and chronic p53 inhibition could contribute to their opposing effects on decitabine’s efficacy in leukemia cells. How the chronic loss of p53 induces these distinct changes in myeloid neoplasms remains to be elucidated. Given that p53 plays crucial roles to maintain genomic and epigenetic homeostasis^32^, long-term p53 deficiency may induce secondary genetic and/or epigenetic changes that increase the response of MDS/AML cells to decitabine. Key factors involved in these changes need to be identified in future studies.

Our study also revealed that p53 plays a critical role to regulate therapeutic resistance in MLL-fusion-driven AML and mutant ASXL1-driven MDS/AML. Although *TP53* mutations rarely coexist with MLL-fusions and *ASXL1* mutations, nonmutational p53 dysfunction could contribute to the development of myeloid neoplasms with MLL-rearrangements or ASXL1 mutations. Indeed, several studies have shown that MLL-fusion oncoproteins suppress p53 function^33–35^. Furthermore, we previously showed that mutant ASXL1 derepressed miR-125a^18^, which was shown to downregulate p53 expression^36^. We also found that the MDM2 inhibitor DS-5272 has potent antileukemia effects against MLL-AF9 leukemia (Hayashi Y *et al*., unpublished data). These findings suggest that multiple nonmutational mechanisms can disturb p53 function in these myeloid neoplasms. In addition to the well-known somatic *TP53* mutations, germ line mutations of *TP53* occur in a small fraction of AML patients and are particularly frequent in therapy-related AML^37^. The MLL-AF9-driven leukemia model using *Trp53*(−/−) cells could be a good experimental model for AML with germ line *TP53* mutations. Our data also raise the possibility that AML patients with germline *TP53* mutations, who often show resistance to chemotherapies, might respond well to decitabine treatment.

In summary, we showed the opposing effects of acute and chronic p53 inhibition on decitabine’s efficacy in MDS and AML cells. Acute inhibition of p53 confers resistance to decitabine, while long-term chronic inhibition of p53 increases sensitivity to decitabine in myeloid neoplasms. Our results imply that the favorable clinical response of patients with *TP53* mutations to decitabine is not the direct consequence of p53 dysfunction, but is likely due to secondary genetic/epigenetic alterations provoked by p53 dysfunction in MDS/AML cells.

## Materials and Methods

### Plasmids and Viral transduction

We used pMSCV-MLL-AF9-pgk-EGFP for MLL-AF9 expression^22^. T7-p53DD-pcDNA3 (Addgene #25989) was obtained from Addgene, and we cloned it to pMYs-IRES-NGFR vector. Retroviruses were generated by transient transfection of Plat-E packing cells with the calcium-phosphate coprecipitation method^38^. Retrovirus transduction to the cells was performed using Retronectin (Takara Bio Inc., Otsu, Shiga, Japan). Lentiviruses were generated by cotransfection of 293T cells with viral plasmids along with pMD2G-VSVG (Addgene #12259) and PAX2 packaging plasmid (Addgene #12260) using the calcium-phosphate coprecipitation method^39^.

### Cell culture

MLL-AF9 cells were cultured in MethoCult™ M3234 (STEMCELL Technologies, Bancouber, BC, Canada) or IMDM medium supplemented with 20% fetal bovine serum, with 10 ng/ml mouse stem cell factor (SCF), 10 ng/ml mouse granulocyte macrophage colony-stimulating factor (GM-CSF), 10 ng/ml mouse interleukin-3 (IL-3) and 10 ng/ml mouse interleukin-6 (IL-6) (R&D Systems, Minneapolis, MN). cSAM and cRAM cells were cultured in RPMI-1640 medium supplemented with 10% fetal bovine serum and 1 ng/ml IL-3. 293 T cells were cultured in DMEM media containing 10% FBS.

### *Trp53* depletion using CRISPR/Cas9

To generate sgRNA expression vectors targeting *Trp53*, annealed oligonucleotides were cloned into the lentiGuide-Puro vector (Addgene #52963) or pLKO5.sgRNA.EFS.tRFP657 vector (Addgene #57824). The expression vector for Cas9 (lentiCas9-Blast #52962) was obtained from Addgene. MLL-AF9, cSAM and cRAM cells were infected with the virus for 24 h, and were selected for stable expression of Cas9 using blasticidin (10 μg/ml). For stable expression of puromycin-resistant sgRNAs, sgRNA-transduced cells were selected using puromycin (1 μg/ml) in M3234 (STEMCELL Technologies) methylcellulose containing 20 ng/ml SCF, 10 ng/ml GM-CSF, 10 ng/ml IL-3 and 10 ng/ml IL-6 (for MLL-AF9 cells) or RPMI-1640 medium supplemented with 10% fetal bovine serum and 1 ng/ml IL-3 (for cSAM and cRAM cells). Sequences for the nontargeting (NT) control and sgRNAs targeting *Trp53* are provided as follows: NT: 5′ cgcttccgcggcccgttcaa 3′, sgTrp53-(1): 5′ gaagtcacagcacatgacgg 3′, sgTrp53-(2): 5’ aacagatcgtccatgcagtg 3′.

### Mice

C57BL/6 (Ly5.2) mice (Sankyo Labo Service Corporation, Tokyo, Japan) were used for bone marrow transplantation assays. *Trp53*(−/−) mice, in which 5′ part of exon 2 including translation initiation site of *Trp53* gene was replaced with Neomycin resistance gene, were provided from the RIKEN BioResource Center (Ibaragi, Japan)^40^. All animal studies were approved by the Animal Care Committee of the Institute of Medical Science at the University of Tokyo (approval number: PA13-19, PA16-31, PA17-75), and were conducted in accordance with the Regulation on Animal Experimentation at University of Tokyo based on International Guiding Principles for Biomedical Research Involving Animals.

### Transplantation assay and Decitabine treatment

Mouse bone marrow cells derived from wild-type or *Trp*53(−/−) mice were transduced with MLL-AF9, and were transplanted intravenously into sublethally irradiated (525 cGy) recipient mice. MLL-AF9-expressing leukemia cells were harvested from spleens of moribund mice, and were serially transplanted into recipient mice. The serial transplantation was subsequently repeated several times to generate MLL-AF9 cells with strong leukemogenicity. The MLL-AF9 cells were then transduced with Cas9 and sgRNAs, or transduced with vector or p53DD, and were injected intravenously into non-irradiated recipient mice (5 × 10^5^/body). For experiments in Fig. 4, wild-type and *Trp*53(−/−) MLL-AF9 cells (1–5 × 10^5^ cells/body) were injected intravenously into non-irradiated recipient mice. For competitive transplantation, we transduced mCherry into wild-type MLL-AF9 cells as a marker, and mixed wild-type and *Trp*53(−/−) MLL-AF9 cells before transplantation. For treatment with decitabine, mice were subcutaneously administered with vehicle control or decitabine (0.6 mg/kg/day) every other day from day 1. Decitabine (HY-A0004) was purchased from MedChemexpress.

### Human cord blood cell culture

Human cord blood (CB) were obtained from the Japanese Red Cross Kanto-Koshinetsu Cord Blood Bank (Tokyo, Japan). Mono nuclear cells (MNCs) were isolated from CB by density gradient centrifugation using Lymphoprep^TM^ (density 1.077; Alere Technologies AS, Oslo, Norway). The CD34^+^ cell fraction was then isolated from the MNCs using the MidiMACS system (CD34^+^ Microbead Kit; Miltenyi Biotec; Bergisch Gladbach, Germany) according to the manufacturer’s protocols. CB CD34^+^ cells were incubated in StemSpan^TM^ SFEMII (STEMCELL Technologies) supplemented with 50 ng/ml mouse Flt-3, 50 ng/ml human TPO, 50 ng/ml human SCF, 25 ng/ml human IL-6 (R&D Systems) and 750 nM stemregenin-1^41^.

### Cell viability assay

Cells were seeded in a 96-well plate at a density of 1 × 10^4^ cells per well, and were cultured with DS-5272 or decitabine for 72 h. Cell viability was measured by Cell Counting Kit-8 (Dojindo, Kumamoto, Japan) according to the manufacturer’s protocols. The absorbance were measured with Wallac 1420 ARVOsx (PerkinElmer Co., Ltd.).

### Cell cycle and apoptosis analyses

For cell cycle and apoptosis analyses, cells were treated with 50 nM decitabine for 6 or 24 hours, respectively. Cell cycle analysis (CycletestTM Plus DNA Reagent Kit; BD Biosciences, San Jose, CA, USA, catalog #340242) and apoptosis analysis (APC Annexin V; BD Biosciences, San Jose, CA, USA, catalog #550474) were performed according to the manufacturer’s protocols using FACS Verse (BD Bioscience, San Jose, CA, USA). The data were analyzed using FlowJo software (Treestar, Inc., San Carlos, CA).

### Western blot analysis

MLL-AF9 cells, cSAM cells and cRAM cells were lysed in SDS sample buffer (125 mM Tris-HCl pH 6.8, 4% SDS, 20% glycerol, 0.01% bromophenol blue, 10% 2-mercaptoethanol). Whole-cell lysates were subjected to sodium dodecyl sulfate-polyacrylamide gel electrophoresis and transferred to a polyvinylidene fluoride membrane (Bio-Rad). The blot was incubated with anti-p53 antibody (#2524; Cell Signaling Technology, Beverly, MA) and anti-GAPDH antibody (#5174; Cell Signaling Technology, Beverly, MA). For LC3 expression, sgTrp53-transduced and *Trp53*-deficient MLL-AF9 cells were treated with 50 nM decitabine or DMSO for 24 hours and were lysed in sample buffer (2 × Laemmli Sample Buffer, #1610737; Bio-Rad). The blot was incubated with anti-LC3 antibody (sc-376404; Santa Cruz Biotechnology) and anti-GAPDH antibody (#5174; Cell Signaling Technology, Beverly, MA). Signals were detected with ECL Western Blotting Substrate (Promega, Madison, WI, USA) and visualized with imagequant LAS 4000 (Fujifilm Life Science, Roche Diagnostics) or Amersham Imager 600 (GE Healthcare). Band intensity was measured using ImageQuant TL Version 8.1 software (GE Healthcare).

### Flow cytometry

Peripheral blood was obtained from leukemic mice treated with vehicle or decitabine. After removing red blood cells using RBC lysis buffer, expression of GFP and tRFP were analyzed by a FACS Verse (BD Biosciences, San Jose, CA, USA). The sgTrp53-transduced and *Trp53*-deficient MLL-AF9 cells were treated with 50 nM decitabine or DMSO for 24 hours and were stained by fluoro-conjugated antibodies [anti-mouse CD117 (BioLegend, catalog #105808, clone 2B8, 1:400), and anti-mouse Gr-1 (BioLegend, catalog #108411, clone RB6-8C5, 1:400)] for 30 min at 4 °C. After staining, cells were washed with cold PBS several times, and were resuspended with PBS containing 2% FBS. Cells were analyzed on a FACS Verse (BD).

### Quantitative PCR

24 hours after 50 nM decitabine or DMSO treatment, total RNA was extracted from sgTrp53-transduced and *Trp53*-deficient MLL-AF9 cells using the RNeasy Mini kit (QIAGEN), and reverse-transcribed using the High Capacity cDNA Reverse Transcription Kit (Applied Biosystems, Foster City, CA, USA) with the deoxyribonuclease I (Invitrogen - Thermo Fisher Scientific - MA, USA). Quantitative PCR (qPCR) was performed using SYBR Premix EX Taq (Takara Bio) and Rotor-Gene Q (Qiagen, Venlo, The Netherlands). Sequences of the primers used for qPCR in this study, from 5′ to 3′ are as follows:

Bax Fw: TGAAGACAGGGGCCTTTTTG, Bax Rv: AATTCGCCGGAGACACTCG, Bbc3 Fw: TGTCGATGCTGCTCTTCTTG, Bbc3 Rv: GTGTGGAGGAGGAGGAGTGG, Cdkn1a Fw: ATCACCAGGATTGGACATGG, Cdkn1a Rv: GTGTGGAGGAGGAGGAGTGG, Fas Fw: TATCAAGGAGGCCCATTTTGC, Fas Rv: TGTTTCCACTTCTAAACCATGCT, Gls2 Fw: CGTCCGGTACTACCTCGGT, Gls2 Rv: TGTCCCTCTGCAATAGTGTAGAA, Prkab1 Fw: AGGCCCAAGATCCTCATGGA, Prkab1 Rv: GGGGGCTTTATCATTCGCTTC, Sesn1 Fw: GGCCAGGACGAGGAACTTG, Sesn1 Rv: AAGGAGTCTGCAAATAACGCAT, Sesn2 Fw: TCCGAGTGCCATTCCGAGAT, Sesn2 Rv: TCCGGGTGTAGACCCATCAC, Gapdh Fw: TTGATGGCAACAATCTCCAC, Gapdh Rv: CGTCCCGTAGACAAAATGGT.

### Morphological analysis

Cytospin preparations were stained with Wright-Giemsa. Images were obtained with a BX51 microscope and a DP12 camera (Olympus).

### Statistics

Statistical significance was determined by the indicated tests for independent variables using GraphPad Prism 7 (GraphPad Software Inc., La Jolla, CA). Statistical analyses for evaluating differences between two groups were performed by Sidak’s multiple comparisons test or Mann-Whitney U test. The survival distributions were compared by the log-rank test. No specific statistical methods were used to predetermine the sample size.

## Supplementary information

Supplemental information

## Data Availability

All data generated or analyzed during this study are included in this published article. The datasets generated during the current study are available from the corresponding author on reasonable request.
