# Supplementary material for: Opposing effects of acute versus chronic inhibition of p53 on decitabine’s efficacy in myeloid neoplasms
Source: Sci Rep. 2019 Jun 3;9:8171. doi: 10.1038/s41598-019-44496-6 (PMC6547685; doi:10.1038/s41598-019-44496-6)

Supplemental Information

Opposing effects of acute versus chronic inhibition of p53 on decitabine's efficacy in myeloid neoplasms

Moe Tamura[1], Taishi Yonezawa[1], Xiaoxiao Liu[1], Shuhei Asada[1], Yasutaka Hayashi[1], Tomofusa Fukuyama[1], Yosuke Tanaka[1], Toshio Kitamura[1], Susumu Goyama[1*]

[1]Division of Cellular Therapy, The Institute of Medical Science, The University of Tokyo, 4-6-1 Shirokanedai, Minato-ku, Tokyo 108-8639, Japan

*Corresponding author

Supplemental Figure 1
Supplemental Figure 2
Supplemental Figure 3
Supplemental Figure 4

Supplemental Figure 1

Full blots for Figure 1c

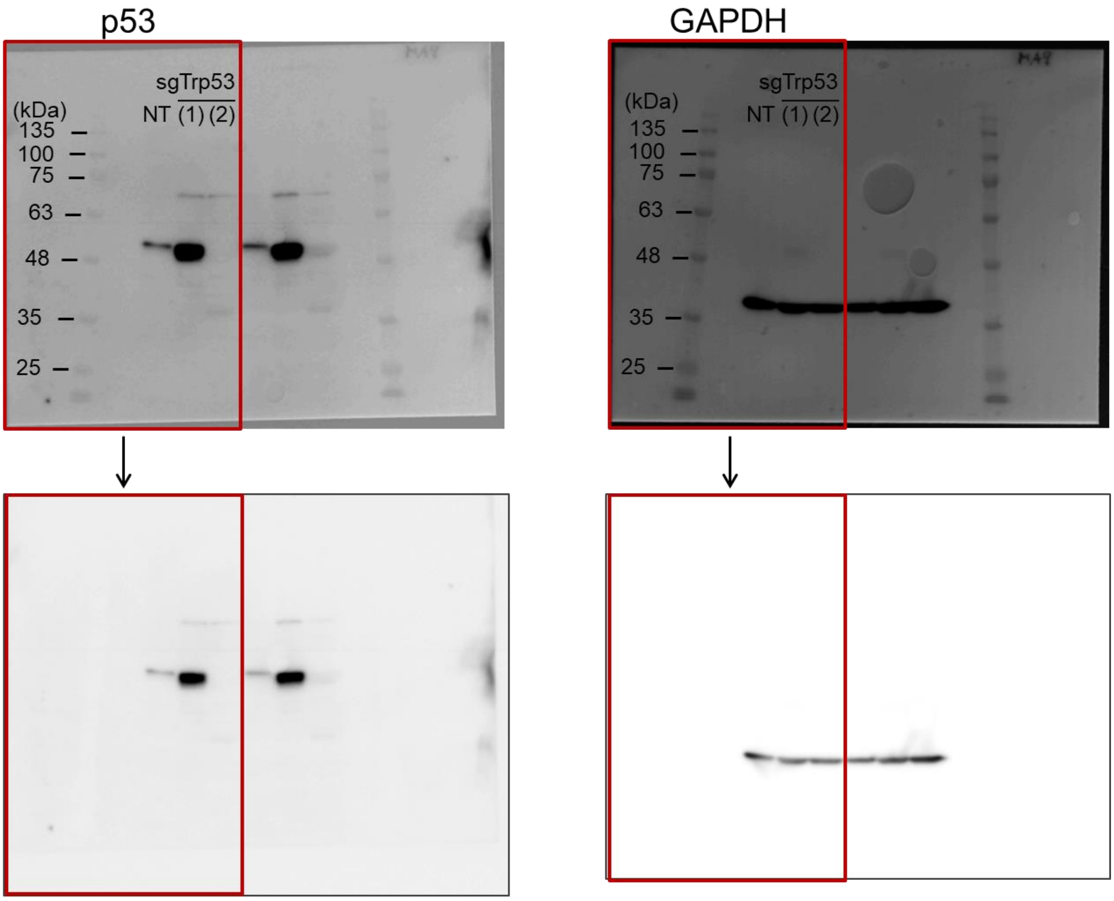

Supplemental Figure 2

Full blots for Figure 3b cSAM/Cas9 cells

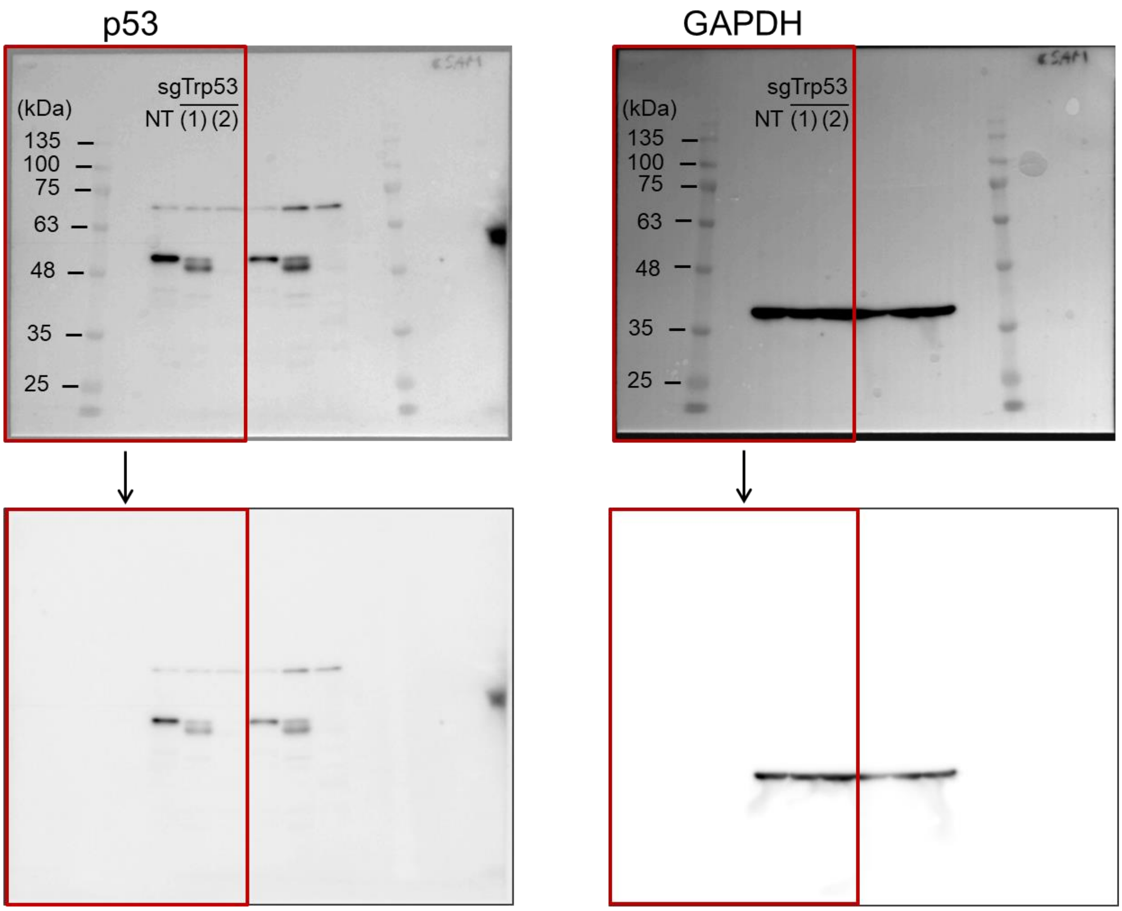

Supplemental Figure 3

Full blots for Figure 3b cRAM/Cas9 cells

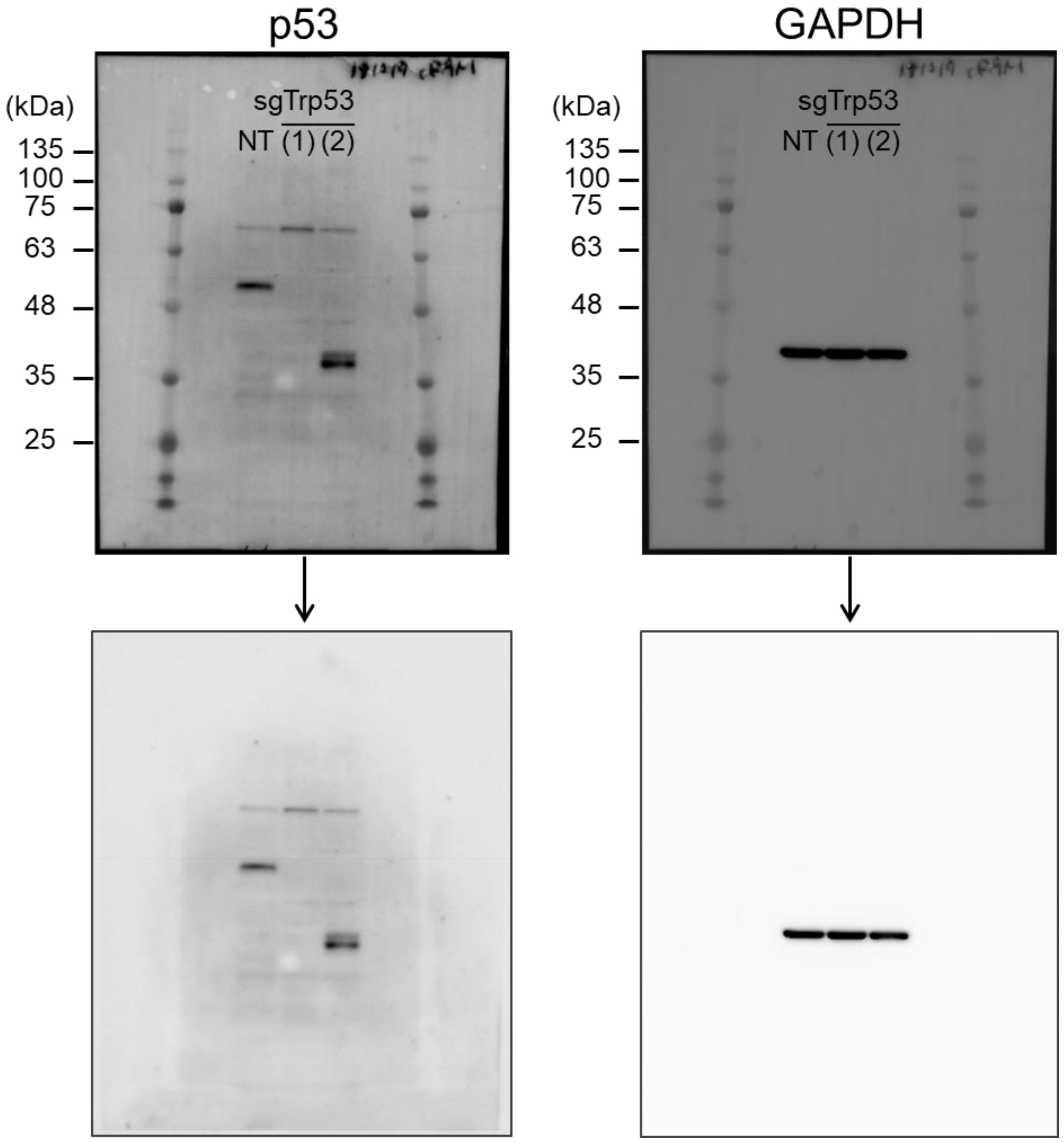

Supplemental Figure 4

Full blots for Figure 7d

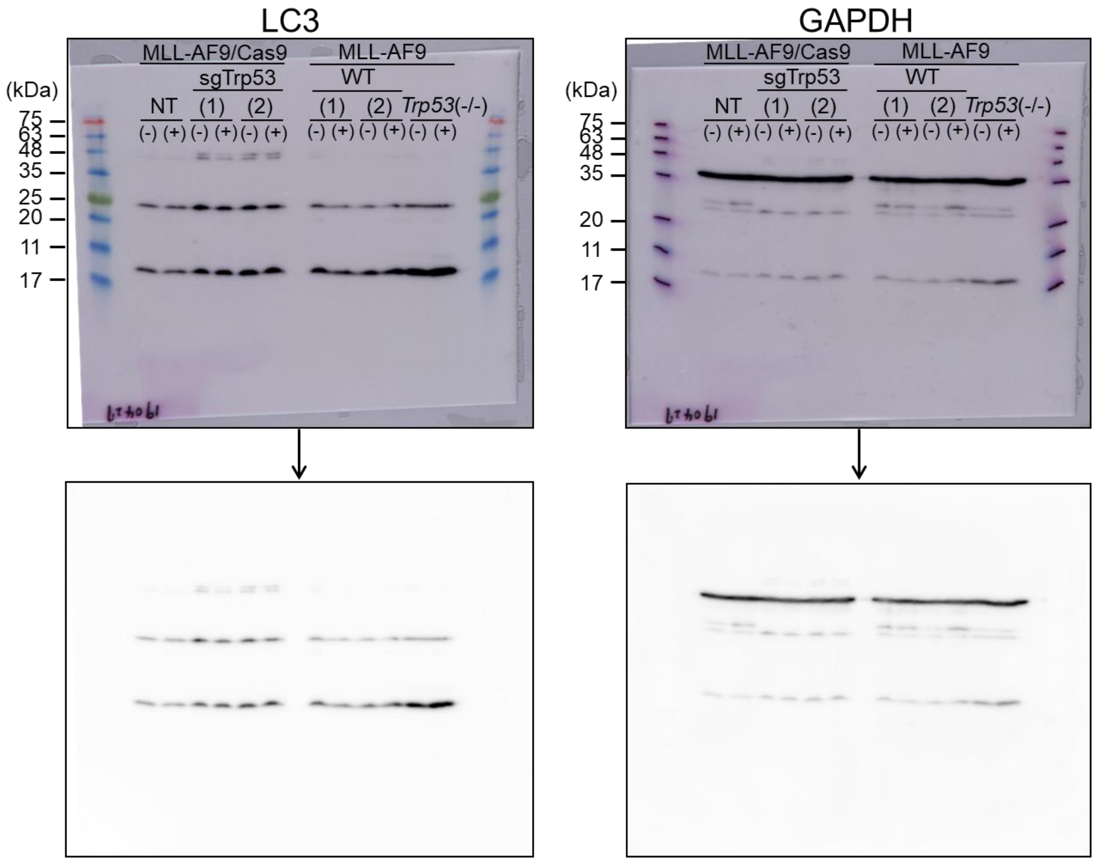

Supplement: Supplementary file 1 — Supplemental information [file 41598_2019_44496_MOESM1_ESM.pdf]
